# Cyclooxygenase-2 expression in the tumor environment is associated with poor prognosis in colorectal cancer patients

**DOI:** 10.3892/ol.2013.1426

**Published:** 2013-06-26

**Authors:** PENG-CHAN LIN, YIH-JYH LIN, CHUNG-TA LEE, HSIAO-SHENG LIU, JENQ-CHANG LEE

**Affiliations:** 1 Department of Internal Medicine, College of Medicine, National Cheng Kung University, Tainan 704, Taiwan, R.O.C.; 2 Department of Surgery, College of Medicine, National Cheng Kung University, Tainan 704, Taiwan, R.O.C.; 3 Department of Pathology, College of Medicine, National Cheng Kung University, Tainan 704, Taiwan, R.O.C.; 4 Department of Microbiology and Immunology, College of Medicine, National Cheng Kung University, Tainan 704, Taiwan, R.O.C.; 5 Center for Gene Regulation and Signal Transduction Research, National Cheng Kung University, Tainan 704, Taiwan, R.O.C.

**Keywords:** COX-2, tumorigenesis, colorectal cancer

## Abstract

The development of colorectal cancer (CRC) is commonly accompanied by the overexpression of the cyclooxygenase-2 (COX-2) gene, with high levels being most common in early colorectal lesions. In the present study, we hypothesized that the expression of COX-2 in normal mucosa affects the expression of COX-2 in adjacent tumors. COX-2 protein expression levels were determined in tumor tissues and the adjacent normal mucosa of 49 paired clinical CRC specimens using western blotting and immunohistochemistry (IHC) staining. The majority of specimens exhibited an extremely low level of COX-2 expression in the tumor tissue and a markedly higher expression level in the adjacent normal tissue, however, high COX-2 expression in the tumor was shown to correlate with a high recurrence rate and poor overall survival. Of the nine CRC cell lines, HT29 showed consistently higher levels of COX-2 expression. Therefore, COX-2 expression in the normal tissue adjacent to the tumor may be involved in the tumorigenesis of CRC. These observations are likely to be useful in determining the significance of COX-2 expression in the tumorigenesis of CRC.

## Introduction

Colorectal cancer (CRC) is the leading cause of cancer-related morbidity and mortality in Taiwan, with ~10,000 new cases and 4,200 mortalities reported each year. Colon cancer progresses via a multistep process known as the adenoma to carcinoma sequence, which has histological and molecular consequences (1). Over 140 years ago, the German pathologist, Rudolf Virchow hypothesized that chronic colonic inflammation was a risk factor predisposing individuals to colon carcinogenesis (2,3). During chronic inflammation, constitutive cellular activation and release of proinflammatory factors damages otherwise healthy neighboring epithelial cells, promoting carcinogenesis by damaging targets and pathways crucial for normal tissue homeostasis (4).

Marked cyclooxygenase-2 (COX-2) expression is detected in cancer and inflammatory cells, the vascular endothelium and fibroblasts of the cancer lesions. COX enzymes produce a number of substances, including prostaglandins (bioactive lipid molecules), that function as major effectors of cancer initiation and progression (5–7). It is widely accepted that the deregulation of the COX-2 signaling pathway affects colorectal tumorigenesis. COX-2 is commonly overexpressed in early neoplastic lesions in the colon and rectum and its expression has been shown to correlate with cell proliferation, differentiation, tumorigenesis and the inhibition of the mitochondrial apoptotic pathway (8). The mechanism of COX-2 induction in these tumors is not fully understood, however, COX-2 expression may be stimulated by proinflammatory cytokines, growth factors, tumor promoters or mutagenic substances under inflammatory and tumor growth conditions (9,10).

A number of previous studies have identified that COX-2 protein expression is higher in normal colonic mucosa than in tumor tissue (6,11). However, by contrast, other studies have demonstrated that COX-2 expression is absent in normal colonic mucosa but high in tumor tissue, and that the long-term use of non-steroidal anti-inflammatory drugs lowers the risk of developing CRC by 40–50% (12). The mechanism underlying the effect of COX-2 on tumor growth has not been determined, but it is hypothesized that stromal and tumor-derived COX-2 affect tumor angiogenesis and/or immune function (13). In the current study, COX-2 expression in tumor tissue and the adjacent normal mucosa were compared to define the extent of COX-2 expression in the tumor microenvironment.

Peroxisome proliferator activated receptor γ (PPAR-γ) functions as a nuclear receptor with antitumor and anti-inflammatory effects. It has been hypothesized that the majority of PPAR-γ is restricted to adipose tissue and that its activation inhibits the nuclear translocation of nuclear factor (NF)-κB (14). Numerous studies have shown that the PPAR-γ ligand has a therapeutic effect on colitis and an antineoplastic effect on CRC (15–18). PPAR-γ is highly expressed in normal colonic mucosa, colon cancer cell lines and tumors (19).

In the present study, we hypothesized that the expression of COX-2 in the normal mucosa affects the expression of the COX-2 gene in the adjacent tumor tissue. A total of 49 pairs of CRC tissues and adjacent normal mucosa specimens were investigated for COX-2 and PPAR-γ expression and the correlation between COX-2 and PPAR-γ expression and survival rate was evaluated. In addition, nine colon cancer cell lines were investigated.

## Materials and methods

### Patients

To determine the levels of COX-2 and PPAR-γ expression in human CRC tissue and adjacent normal tissue (5 cm from the tumor margin), 49 specimen pairs (98 specimens) were evaluated by immunohistochemistry (IHC) and western blot analysis. The samples were obtained from patients who had received curative surgery for early-stage, primary CRC at the National Cheng Kung University Hospital (Tainan, Taiwan) between January 2000 and December 2001. Patient characteristics are shown in Table I. This study was approved by the Institutional Review Board of The National Cheng Kung University Hospital (Tainan, Taiwan).

### Cell lines

Cell lines derived from human colon carcinomas at various stages were purchased from American Type Culture Collection (ATCC; Manassas, VA, USA). HT29 cells (grade I colorectal adenocarcinoma), HT116 cells (colorectal carcinoma) and Daudi cells (B lymphoblasts) were maintained in DMEM with 10% fetal bovine serum (FBS). Caco2 (colorectal adenocarcinoma) and T84 (metastatic carcinoma) cells were maintained in DMEM with 20 and 5% FBS, respectively. SW116 (Dukes A), SW480 (Dukes B) and SW620 (Dukes C) cells (all from colorectal adenocarcinomas) were maintained in L-15 medium with 10% FBS. C205 (Dukes D) cells (colorectal adenocarcinoma and ascites metastasis) were maintained in RPMI-1640 medium with 10% FBS.

### IHC

IHC was performed as described previously (20). Tissue sections were incubated at room temperature (RT) for 2 h with monoclonal antibodies against COX-2 and PPAR-γ (Thermo Fisher Scientific, Cheshire, UK). The optimal dilution (1:100–1:200) was determined using human kidney tissue as a positive control. The StrAviGen Super Sensitive MultiLink kit (BioGenex Laboratories, Inc., San Ramon, CA, USA) was used to detect the resulting immune complex. Peroxidase activity was visualized using an aminoethyl carbazole substrate kit (Zymed Laboratories, Inc., San Francisco, CA, USA). Sections were counterstained with hematoxylin and non-immune mouse immunoglobulin was used in place of the primary antibody to serve as a control. Since no significant differences in staining intensity were identified, only the proportion of tumor cells that were stained was evaluated. The staining of COX-2 and PPAR-γ was scored as negative if <10% of the tumor cells showed membranous immunoreactivity (21).

### Western blot analysis

The cells were lysed with WCE buffer containing 20 mM 2-[4-(2-hydroxyethyl)piperazin-1-yl]ethanesulfonic acid (pH 7.9), 5% octylphenoxypolyethoxyethanol CA-630, 7.5% glycerol, 150 mM NaCl, 1 mM EDTA, 210 μg/ml NaF, 1 mM Na_3_VO_4_, 1 mM dithiothreitol, 1 μg/ml leupeptin, 1 μg/ml pepstatin, 1 μg/ml aprotinin and 0.5 mM phenylmethanesulfonylfluoride. For the western blot analysis, proteins were resolved in an 8–12% SDS-PAGE gel and electrotransferred to a polyvinylidene fluoride membrane according to standard procedure. Following blocking for 1 h with 5% skimmed dry milk in TBS-T buffer (2.4 g Tris, 8.8 g NaCl and 1 ml Tween 20) dissolved in 1 l deionized H_2_O (pH 7.4), the blot was probed with the primary antibodies overnight at 4°C. Next, the blot was incubated with peroxidase-conjugated secondary antibody for 1 h at RT followed by detection of the protein with enhanced chemiluminescence reagents and exposure to X-ray film.

### Statistical analysis

Statistical significances between COX-2 and PPAR-γ expression and clinical and pathological parameters were assessed using the χ^2^ or Mann-Whitney U tests. Kaplan-Meier curves were used to assess the effect of COX-2 and PPAR-γ expression on disease-free and overall survival. Overall survival was defined as the time between surgery and patient mortality due to CRC. Individuals who succumbed to additional causes or survived to the last follow-up were censored. All P-values were based on a two-tailed statistical analysis and P<0.05 was considered to indicate a statistically significant difference. The correlation between COX-2 and PPAR-γ was evaluated by linear regression analysis.

## Results

### COX-2 and PPAR-γ expression in colorectal tumor specimens, as determined by western blotting

**Figure 1:**
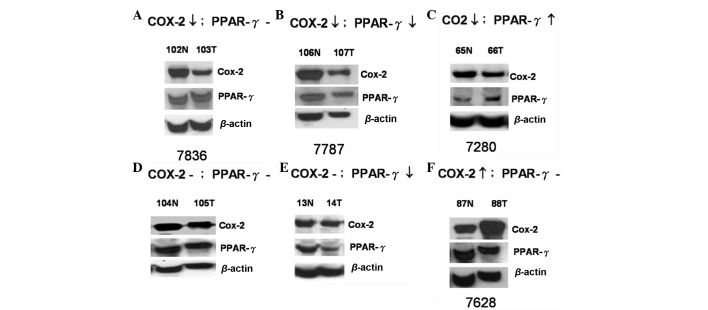
COX-2 and PPAR-γ expression in tissues from colorectal cancer (CRC) patients. Total protein extracted from frozen colorectal tumor and paired colorectal normal tissues were analyzed by western blotting using monoclonal antibodies against COX-2 and PPAR-γ (n=49). β-actin served as the internal control. N, normal tissue; T, tumor tissue; COX-2, cyclooxygenase-2; PPAR-γ, peroxisome proliferator activated receptor γ.

**Figure 2:**
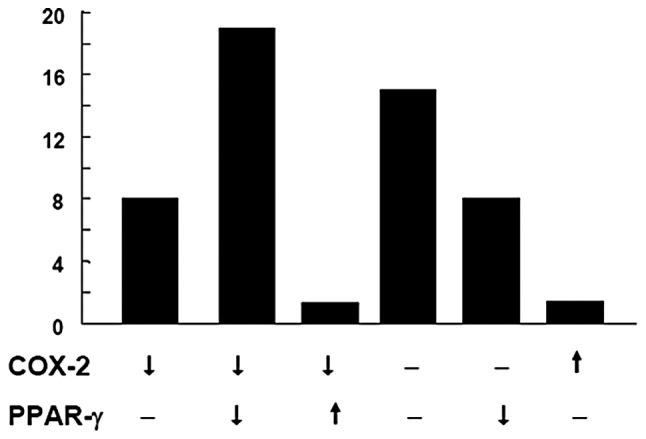
Classification of colorectal cancer (CRC) specimens according to levels of COX-2 and PPAR-γ expression. A gel scanner was used to measure the intensity of the COX-2 and PPAR-γ bands presented in Fig. 1. Levels of COX-2 and PPAR-γ expression in the 49 paired specimens (tumor and normal tissue) were compared and characterized as increased (↑), decreased (↓) and unchanged (−). COX-2, cyclooxygenase-2; PPAR-γ, peroxisome proliferator activated receptor γ.

The levels of COX-2 and PPAR-γ expression in the paired specimens from 49 patients were measured by western blot analysis. The expression profiles were categorized into six groups: i) COX-2 decreased, PPAR-γ unchanged (8; 16.3%); ii) COX-2 decreased, PPAR-γ decreased (18; 36.7%); iii) COX-2 decreased, PPAR-γ increased (1; 2.04%); iv) COX-2 unchanged, PPAR-γ unchanged (14; 28.6%); v) COX-2 unchanged, PPAR-γ decreased (7; 14.3%); and vi) COX-2 increased, PPAR-γ unchanged (1; 2.04%; Fig. 1). The quantified data of the six groups are shown in Fig. 2. In summary, the highest percentage of colon cancer specimens showed decreased expression (18; 36.7%) or no change in expression (14; 28.6%) of COX-2 and PPAR-γ. Only 2.04% of specimens showed increased COX-2 expression in the tumor tissues, which is inconsistent with a previous study (9).

### COX-2 expression in colorectal tumor specimens determined by IHC

**Figure 3:**
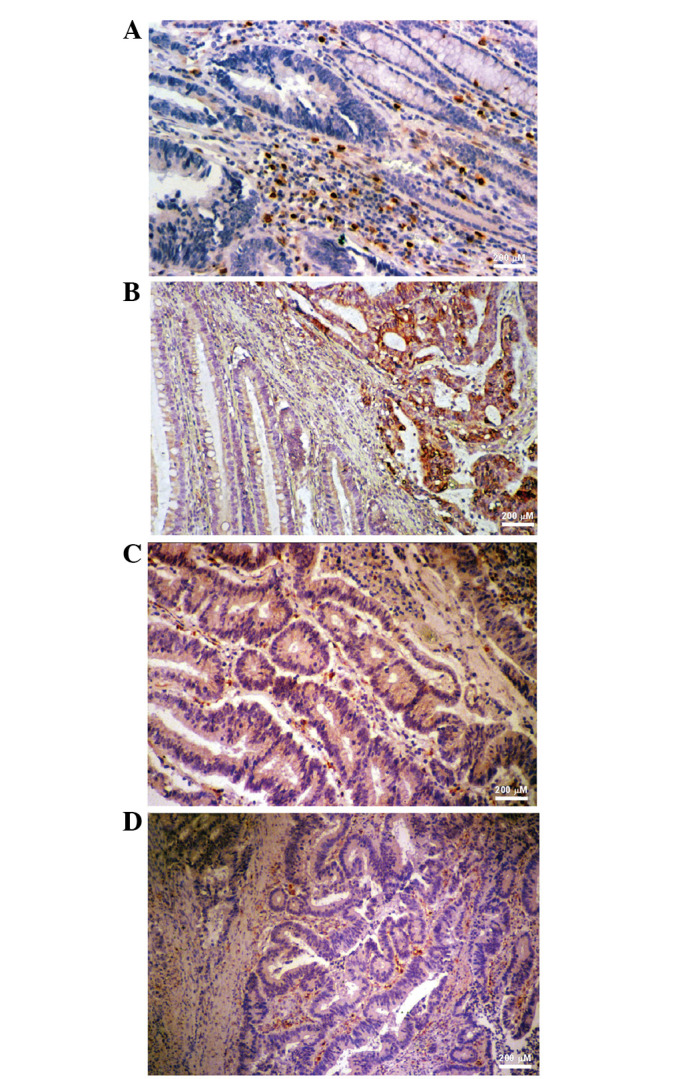
Level of COX-2 expression in colorectal cancer (CRC) specimens, as determined by immunohistochemistry. Paraffin sections of CRC specimens were stained with monoclonal anti-COX-2 antibody followed by peroxidase-conjugated secondary antibody. Brown indicates COX-2 staining. The staining intensity reflects the level of COX-2 expression and four patterns are shown: (A) Extremely low (magnification, ×200) and (B) high (magnification, ×100) COX-2 expression in the tumor tissue compared with adjacent stromal tissue. (C) COX-2 expression in tumor and adjacent stromal tissue (magnification, ×200). (D) Low COX-2 expression in the tumor tissue compared with adjacent stromal tissue (magnification, ×100). COX-2, cyclooxygenase-2.

COX-2 staining was strong in the adjacent stromal cells of specimen #7280, but weak within the tumor tissue (Fig. 3A), which was consistent with the results of the western blot analysis (Fig. 1). IHC of specimen #7628 showed that COX-2 was overexpressed in the gland cells of the tumor tissue but not in the normal and stromal cells (Fig. 3B), which was also consistent with the western blot analysis (Fig. 1). COX-2 staining in specimen #7787 was marked in the gland and stromal cells of the colorectal tumor specimen (Fig. 3C) and COX-2 expression was higher in the normal tissue compared with the tumor tissue (Fig. 1). In specimen #7836, COX-2 expression was higher in the surrounding stromal cells (Fig. 3D) and normal tissue (Fig. 1) than in the tumor tissue, as determined by IHC staining and western blotting, respectively. The majority of results from the current study show a higher expression of COX-2 in the adjacent normal tissues and stromal cells than in the tumor tissue.

### Correlation between COX-2 and PPAR-γ expression

**Figure 4:**
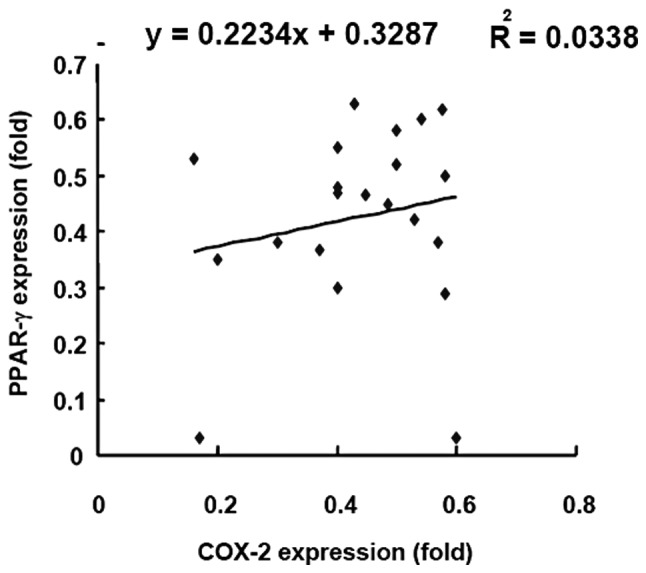
Correlation between the levels of COX-2 and PPAR-γ expression. Fold increase in COX-2 and PPAR-γ (T:N) in a total of 21 paired specimens. The X and Y axes represent the fold increase in COX-2 and PPAR-γ, respectively. Linear regression was conducted to evaluate the correlation between COX-2 and PPAR-γ. T, tumour; N, normal tissue; COX-2, cyclooxygenase-2; PPAR-γ, peroxisome proliferator activated receptor γ.

To investigate the correlation between the expression of COX-2 and PPAR-γ, the expression levels were investigated in specimens from 21 CRC patients by linear regression analysis (Fig. 4). The R-value of the linear regression line was 0.03 indicating that there was no linear correlation between COX-2 and PPAR-γ expression.

### Relative ratio of tumor-to-normal tissue COX-2 expression correlates with high recurrence rate and poor prognosis

**Figure 5:**
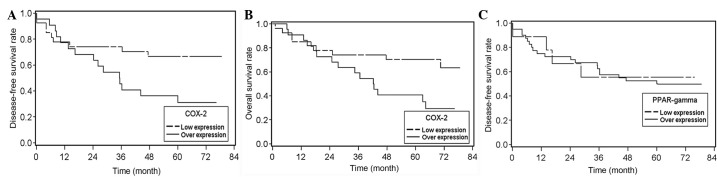
Relative ratio of tumor-to-normal tissue COX-2 expression correlates with disease-free and overall survival in colon cancer patients. High ratio of COX-2 expression correlated with (A) poor disease-free and (B) poor overall survival (P=0.03). (C) No correlation was identified between the ratio of PPAR-γ expression and poor disease-free survival (P=0.23). COX-2, cyclooxygenase-2; PPAR-γ, peroxisome proliferator activated receptor γ.

In the multivariate logistic regression analysis, the recurrence of CRC was identified to significantly correlate with COX-2 expression (tumor tissue vs. normal tissue; P=0.015; n=49; cut-off value, 0.6; Table II). The correlation between COX-2 expression and tumor recurrence was independent of age, gender, histological differentiation, primary tumor origin, tumor size and nodal status, as determined by univariate logistic regression analysis (Table II). High COX-2 expression in the tumor tissues (specimen #7628; Figs. 1 and 3B) also correlated with poor disease-free and overall survival rates. Disease-free and overall survival times were significantly lower in patients with a high tumor-to-normal tissue COX-2 expression ratio when compared with that of subjects with a low tumor-to-normal tissue COX-2 expression ratio (P=0.03; Fig. 5A and 5B). However, no correlation was identified between PPAR-γ expression and disease-free survival (P=0.23; Fig. 5C). In summary, COX-2 overexpression in tumors correlates with recurrence and poor survival, however PPAR-γ overexpression does not.

### Levels of COX-2 and PPAR-γ expression in nine colon cancer cell lines

**Figure 6:**
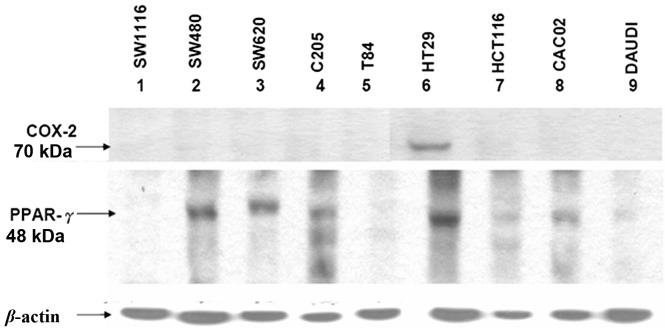
Expression levels of COX-2 and PPAR-γ in nine colon cancer cell lines. Total protein was extracted from each individual cell line and 30 μg protein was loaded onto a 10% SDS PAGE gel. Following electrotransfer of the bands from the gel onto a membrane, monoclonal antibodies against COX-2 and PPAR-γ were used to blot the specific proteins. β-actin served as an internal control. COX-2, cyclooxygenase-2; PPAR-γ, peroxisome proliferator activated receptor γ.

To evaluate the levels of COX-2 and PPAR-γ expression in nine CRC cell lines, namely SW116, SW480, SW620, C205, T84, HT29, HCT116, CACO-2 and DAuD1, representing various grades of malignancy, the total protein extracted from these lines was evaluated by western blotting using monoclonal anti-COX-2 and -PPAR-γ antibodies (Fig. 6). One colon cancer cell line, HT29, expressed COX-2. By contrast, PPAR-γ expression varied in the nine cancer cell lines. The expression of PPAR-γ was high in four of the colon cancer cell lines, while SW480, SW620, C205 and HT29 were demonstrated to be have insignificant or undetectable expression in five. Overall, the majority of the CRC cell lines expressed extremely low levels of COX-2, which was consistent with the results from the CRC patients (Fig. 1).

## Discussion

In the present study, the majority of the patients with colon cancer exhibited low levels of COX-2 expression in the tumor tissues and high levels of COX-2 expression in the adjacent normal tissues, as determined by western blotting and IHC staining. However, a high ratio of tumor-to-normal tissue COX-2 expression was shown to correlate with high recurrence rates and poor prognosis. In addition, previous studies have shown that tumor stromal cells contribute to COX-2 expression in CRC, indicating that normal and tumor cells may contribute to an increase in prostaglandin levels within the tumor microenvironment and the subsequent development of cancer (22). Previously, Charalambous *et al* reported that COX-2 expression in stromal cells correlates with the clinical severity of CRC (11). In general, COX-2 is not detectable in normal and premalignant colorectal epithelium and it has been hypothesized to be confined to subepithelial cells, including fibroblasts, in non-malignant colonic tissue. Fibroblasts and additional mesenchymal cells, including stromal cells, are the source of COX-2 in normal and premalignant colorectal tissues. The moderately higher rate of COX-2 transcription in fibroblasts leads to a corresponding increase in prostaglandin E2 synthesis. The effect of prostaglandin E2 is amplified progressively via the robust stabilization of COX-2 mRNA (22). Intestinal epithelial cells with high expression levels of the COX-2 gene have altered adhesion properties, resist apoptosis and exhibit a marked decrease in retinoblastoma kinase activity, which correlates with the activation of cyclin-dependent kinase 4 (23). Carcinogenesis has previously been reported to correlate with the transformation of normal stroma into a ‘reactive’ stromal phenotype (24). In the current study, COX-2 expression was extremely low in ~75% of tumor tissues and higher in the stromal cells of adjacent normal tissues. The COX-2 expression of cancer cells *in vivo* may be affected by the microenvironment of the tissue surrounding the tumors. Prostaglandin I2 production by stromal cells promotes the survival of colonocytes through PPAR-γ activation. This mechanism may aid the maintenance of cells in normal crypts and the clonal expansion of mutant colonocytes during tumorigenesis (22). In the present study, of the nine colon cancer cell lines representing various grades of malignancy, only HT29 showed increased COX-2 expression, indicating that expression is negatively regulated in the majority of CRC cell lines. However, the underlying mechanism remains unclear. Higher COX-2 expression in the microenvironment adjacent to the tumor may affect the expression of COX-2 in the tumor cells.

The majority of colorectal adenomas and carcinomas are characterized by chromosomal instability and a progressive loss of heterozygosity. By contrast, in 15–20% of colorectal neoplasms, induction occurs via a distinct genetic pathway characterized by microsatellite instability and loss of expression of a DNA mismatch repair enzyme, commonly hMLH1 or hMSH2 (25). Overall, the results of the present study show that 33% of defective mismatch repair was identified in colorectal tumors with low or absent COX-2 staining (P<0.05). Additional features have also been identified to be predictive of low COX-2 staining, including marked infiltration of the tumor by lymphocytes and solid/cribriform or signet ring histological patterns (25). These investigations indicate that CRC with molecular and phenotypic characteristics of defective DNA mismatch repair express lower levels of COX-2. The clinical implications of this biological distinction remain unknown, but must be considered when investigating the efficacy of COX-2 inhibitors for chemoprevention in patients whose tumors may arise in the setting of defective DNA mismatch repair (25).

The growth and differentiation of colon cancer cells are also modulated by PPAR-γ. PPARs are transcription factors that regulate molecular events in normal and cancer cells (26). A number of COX enzymes produce specific eicosanoids that have previously been shown to activate transcription mediated by PPAR-γ. The expression of PPAR-γ is largely restricted to adipose tissue and a marked increase in PPAR-γ RNA levels has been identified in colon tumors compared with paired normal mucosa. PPAR-γ protein expression has been previously reported in 4/5 colon tumor samples (27).

However, the levels of PPAR-γ expression in the nine colon cancer cell lines of the present study were variable. The patterns of COX-2 and PPAR-γ expression in the colon cancer patients were classified into six types and the majority of the specimens showed decreased or unchanged expression levels of COX-2 and PPAR-γ. However, one specimen showed increased expression of COX-2 with unchanged expression of PPAR-γ, whilst a second showed increased expression of PPAR-γ with unchanged expression of COX-2. In addition, no linear correlation between COX-2 and PPAR-γ expression was identified in the 21 colon cancer specimens, demonstrating that the expression of COX-2 and PPAR-γ is not essential for colon cancer formation.

The roles of PPAR-γ, COX-2 and p-IκB-α (important molecular targets for colon cancer chemoprevention) in stromal remodeling were investigated by comparing the expression of these molecules in the tumor and surrounding normal colonic mucosa of stromal myofibroblasts, macrophages and endothelial cells. COX-2 expression was upregulated by NF-κB in the stromal myofibroblasts surrounding the colon adenocarcinomas and the expression was identified to markedly correlate with p-IκB-α expression (P<0.001). No correlation between PPAR-γ, COX-2 or p-IκB-α expression and the stage or differentiation status of the adenocarcinomas was identified (24). In addition, no correlation was shown between PPAR-γ and COX-2 expression.

In conclusion, the observations of the current study indicated that COX2 expression in normal tissue adjacent to tumors may be important for colon cancer carcinogenesis, despite the correlation between a higher ratio of tumor-to-normal tissue COX-2 expression and poor prognosis in CRC.

## Figures and Tables

**Table I:** Characteristics of 49 CRC patients.

Characteristics	Value
Age, years	
Median	61
Range	34–75
Performance status, n	
0–1	47
2	2
Gender, n	
Male	27
Female	22
Histological differentiation, n	
Well	10
Moderate	33
Poor	6
Primary tumor origin, n	
Colon-Sigmoid	34
Rectum	15
Tumor status^a^, n	
T1-T2	6
T3-T4	43
Nodal status^a^, n	
0	30
1	15
2	4
Stage^a^, n	
II	30
III	16
IV	3

a American Joint Committee on Cancer Staging.

CRC, colorectal cancer.

**Table II:** Correlation between COX2 expression and various prognostic factors of colorectal cancer patients.

	COX2^a^, n		
		
Variables	≥0.6	<0.6	P-value
Patients	22	27	
Gender			
Male	12	15	0.944
Female	10	12	
Histological differentiation			
Well	5	5	0.883
Moderate	14	19	
Poor	3	3	
Primary tumor origin			
Colon-sigmoid	15	19	0.869
Rectum	7	8	
Tumor status^b^			
T1-2	2	5	0.138
T3-4	20	22	
Nodal status^b^			
0	13	17	0.965
1	7	8	
2	2	2	
Pathological stage			
II	13	17	0.835
III	8	8	
IV	1	2	
Recurrence			
Yes	15	9	0.015
No	7	18	

a Tumor tissue vs. normal tissue. Expression levels detected by western blotting.

b American Joint Committee on Cancer Staging.
